# Safety and impact of the Mediterranean diet in patients with chronic kidney disease: a pilot randomized crossover trial

**DOI:** 10.3389/fnut.2024.1463502

**Published:** 2024-09-04

**Authors:** Yu-Jin Kwon, Young Su Joo, Hae-Ryong Yun, Li Rang Lim, Juyeon Yang, Hye Sun Lee, Hyung-Mi Kim, Hyangkyu Lee, Jung Eun Lee, Ji-Won Lee

**Affiliations:** ^1^Department of Family Medicine, Yongin Severance Hospital, Yonsei University College of Medicine, Yongin, Republic of Korea; ^2^Division of Nephrology, Department of Internal Medicine, Yongin Severance Hospital, Yonsei University College of Medicine, Yongin, Republic of Korea; ^3^Department of Family Medicine, Severance Hospital, Yonsei University College of Medicine, Seoul, Republic of Korea; ^4^Biostatistics Collaboration Unit, Department of Research Affairs, Yonsei University College of Medicine, Seoul, Republic of Korea; ^5^Department of Food and Nutrition, Dongduk Women's University, Seoul, Republic of Korea; ^6^Nutrition R&D Institute, MEDI.SOLA Co., Ltd., Seoul, Republic of Korea; ^7^College of Nursing, Mo-Im Kim Research Institute, Yonsei University, Seoul, Republic of Korea; ^8^Institute for Innovation in Digital Healthcare, Yonsei University, Seoul, Republic of Korea

**Keywords:** chronic kidney disease, Mediterranean diet, Korea, safety, potassium

## Abstract

**Introduction:**

Emerging evidence highlights the potential advantages of the Mediterranean diet (MD) in preserving kidney function and slowing chronic kidney disease (CKD) progression. However, interventional studies on the MD are scarce in East Asian populations.

**Methods:**

This randomized crossover trial aimed to assess the safety and short-term impact of the Mediterranean Proper Optimal Balance (MEDi-POB) diet in Korean patients with stage 3–4 CKD. Kidney function was assessed using the estimated glomerular filtration rate, which was calculated using the CKD Epidemiology Collaboration equation. Fifty patients with CKD were randomly assigned to two groups, each starting with a different 4-week intervention, followed by a 4-week washout period, followed by a switch to the other 4-week intervention. During the MEDi-POB intervention, patients received home delivery of meals twice daily, 5 days a week, while the control intervention comprised a conventional diet. Forty-six patients successfully completed the entire 12-week trial. Paired *t*-tests were conducted to assess mean differences between the two groups. A linear mixed model was used to adjust for sequence and period.

**Results:**

Dietary fat, fiber, and niacin intake were significantly higher following the MEDi-POB diet than following the control diet (*p* = 0.001 for fat, *p* < 0.001 for fiber, and *p* = 0.007 for niacin). The MEDi-POB diet also yielded slightly increased total CO_2_ levels (*p* = 0.043), indicating effective management of metabolic acidosis. Conversely, sodium and copper intake were significantly lower with the MEDi-POB diet (*p* = 0.032 and *p* = 0.037, respectively). Caloric intake increased, but body mass index slightly decreased from baseline after consuming the MEDi-POB diet. Dietary potassium intake exhibited a non-significant increase (*p* = 0.053), and no significant changes in serum (*p* = 0.883) and urine potassium levels (*p* = 0.087) occurred. Kidney function remained well-preserved following the MEDi-POB diet.

**Conclusion:**

These results indicate that the MEDi-POB diet is safe even in patients with advanced CKD, as it does not adversely affect serum and urine potassium levels and helps maintain kidney function.

## 1 Introduction

Chronic kidney disease (CKD) poses a global health challenge, leading to a substantial rise in economic costs for both the healthcare infrastructure and society at large (1). The progression of CKD, which leads to a requirement for dialysis or kidney transplantation, is linked to an increased risk of cardiovascular disease and all-cause mortality (2). Therefore, proactive intervention to slow disease progression and effectively address cardiovascular disease risk factors is indispensable for the pre-dialysis population.

Emerging evidence suggests that adherence to a healthy diet is associated with a lower risk of CKD progression and overall mortality (3). The Mediterranean diet (MD), characterized by a high consumption of fruits, vegetables, whole grains, nuts, beans/legumes, and olive oil, as well as a low intake of red or processed meat, has beneficial effects on several chronic diseases, such as obesity, cardiovascular disease, and various cancers (4, 5). Several observational and dietary intervention studies have revealed the usefulness of the MD in preserving kidney function and delaying CKD progression (6, 7). The Kidney Disease Outcomes Quality Initiative National Kidney Foundation 2020 guidelines for nutrition in CKD have proposed the MD as the preferred diet for patients with CKD, regardless of CKD stage, for its putative favorable effect on body weight, lipid profile, blood pressure, and net acid production (8). However, the characteristic components of an MD, such as fruit-, vegetable-, and nut-rich meals, may conflict with the traditional dietary restrictions of patients with CKD, who are typically cautioned against potassium-rich foods owing to their potential to contribute to hyperkalemia, affect the electrolyte balance, and have an impact on serum acidity (9).

Recent evidence suggests that the MD's high potassium content does not increase the risk of hyperkalemia or negatively affect kidney health in patients with CKD (10). However, the relevant studies did not include patients with advanced CKD, in whom severely reduced kidney function may interfere with potassium excretion (10). Additionally, clinical trials on the relationship between dietary potassium and serum potassium levels or clinical outcomes are sparse, and interventional studies in non-Mediterranean populations, especially East Asians, are limited.

Salt intake is another crucial component of nutrition in the management of CKD. Reducing salt intake helps with the management of blood pressure, prevention of fluid retention, maintenance of the electrolyte balance, and promotion of overall health (11). Therefore, the Kidney Disease Outcomes Quality Initiative guidelines recommend a sodium intake of 2000–2300 mg per day for patients with CKD stage 3 and above (8). The sodium content of the MD can be lowered by focusing on fresh, whole foods, substituting herbs and spices for salt, reducing processed-food consumption, choosing low-sodium dairy options, and using olive oil as the primary fat source (11). Combining the MD principles with specific sodium restrictions can improve patients' nutritional status and manage the progression of CKD.

Given the diversity of dietary cultures across nations and ethnicities, our research team developed a Korean-style Mediterranean Proper Optimal Balance (MEDi-POB) diet with the ideal ratio of macronutrients to increase the life expectancy of Koreans while maintaining the fundamental concept of the MD (12). In previous studies, the MEDi-POB diet was beneficial not only for patients with dyslipidemia, by improving their lipid parameters, but also in reducing cardiovascular risk, by reducing chronic inflammation and insulin resistance, suggesting that it may also be beneficial for patients with preexisting CKD (13, 14).

We aimed to evaluate the short-term effects of the MEDi-POB diet compared to a conventional diet following nutritional advice for CKD in patients with stage 3–4 CKD, with a primary focus on safety in terms of the physiological levels of potassium. This is the first prospective, crossover, randomized trial of Asian patients with pre-dialysis CKD in which the short-term metabolic benefits and safety of the MD are explicitly examined.

## 2 Materials and methods

### 2.1 Study design and patients

This study was a pilot, randomized, open-label, two-arm, crossover trial. The study protocol was approved by the Institutional Review Board of the Yongin Severance Hospital (IRB No. 9-2021-0117) and registered at the Clinical Research Information Service (KCT0006612).

This study was conducted in compliance with the principles of the Declaration of Helsinki. Informed consent was obtained from all participants. Patients were recruited from October 18, 2021 to August 29, 2022 at the Yongin Severance Hospital (Yongin, South Korea). Patients aged 20 years and older who were diagnosed with stage 3–4 CKD but were not undergoing kidney replacement therapy (hemodialysis or peritoneal dialysis) were eligible for enrollment. The following patients were excluded from this study: those undergoing kidney replacement therapy, those with a baseline serum potassium level equal to or higher than 6.0 mEq/L, and those with food allergies (such as an allergy for shellfish, fish, nuts, eggs, meat, tomatoes, wheat, and soy).

### 2.2 Randomization and study protocol

**Figure 1:**
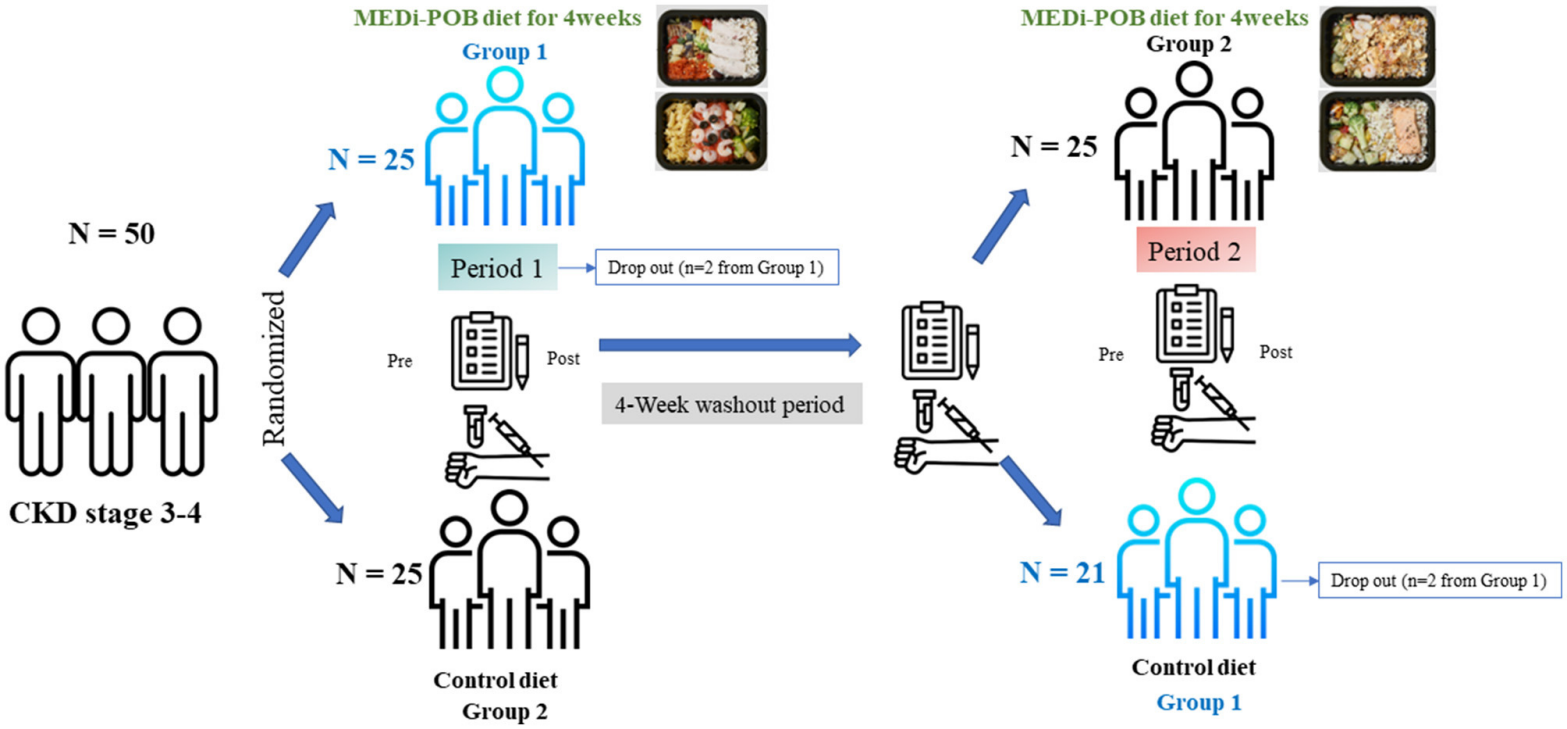
Study scheme.

Patients were randomly assigned to either the MEDi-POB diet or a conventional diet for a duration of 4 weeks. This was followed by a 4-week washout period and subsequently, a second 4-week phase during which the patients switched to the opposite diet (as illustrated in Figure 1). Patients were assigned to the two groups in a 1:1 ratio by using a computer-generated randomization system that was centrally managed. In group 1, patients initially followed the MEDi-POB diet and switched to the conventional diet (sequence 1), while in group 2, patients began with the conventional diet and transitioned to the MEDi-POB diet (sequence 2). During the first intervention phase, two patients in group 1 dropped out, one owing to a urinary tract infection and the other being lost to follow-up. Following the second intervention phase, two patients from group 1 withdrew from the study. Ultimately, 46 patients successfully completed the entire 12-week trial (Figure 1).

### 2.3 Dietary intervention and nutrition education in MEDi-POB and control diets

During the 4-week intervention period, the MEDi-POB group received home-delivered meals from MEDI.SOLA Co., Ltd., Seoul, Korea, twice daily, 5 days a week. The MEDi-POB diet was formulated based on a carbohydrate-fat-protein ratio associated with a decrease in mortality rates among Koreans, as evidenced in previous studies (12). The main characteristic of the MEDi-POB diet is a high intake of vegetables, fruits, and monounsaturated fats from olive oil, with a low intake of saturated fats and meats. The nutritional criteria for the MEDi-POB diet are based on the 2020 Dietary Reference Intakes for Koreans for the general population. The protein intake was set at 0.8 g/kg body weight, corresponding to 46.4–56.8 g in this study. Sodium was limited to within 2,000 mg, as was potassium, ensuring a balanced and controlled meal for boxed lunches. Taking into consideration the importance of sufficient caloric intake for patients with CKD, in addition to the twice-daily home-delivered meals, the study patients were advised to eat half a slice of whole wheat bread and one teaspoon of olive oil for breakfast. As a substitute for snacks, patients were recommended to consume 200 ml of a standard liquid nutritional supplement, 4 g of dietary fiber (partially hydrolyzed maltodextrin), and half an apple per day. Nutritional information on the menus of the MEDi-POB diet and standard liquid nutritional supplement are provided in Supplementary Tables 1, 2.

For the MEDi-POB diet, patients received the following nutritional education: (1) recommendation to consume complex carbohydrates (mixed grain rice or whole grain bread), (2) limitation of intake of red meat and processed meat products, and encouragement of consumption of fish, seafood, beans, or tofu, (3) encouragement of fruit and vegetable consumption, (4) recommendation to use olive oil and sesame oil, (5) suggestion to reduce consumption of beverages and snacks high in simple sugars, and (6) promotion of snacks in the form of fruits and nuts.

For the control diet, patients received the following nutritional education, in line with the conventional guidelines for patients with advanced CKD: (1) instruction that proteins should be consumed in portions of no more than two servings per day, (2) instruction to choose foods low in potassium, phosphorus, and sodium (guidance was provided on restricted and suitable foods), (3) cooking tips to reduce potassium intake, and (4) recommendation to consume MEDi-POB meals with equivalent calories. Qualified nutritionists collected information from the patients regarding all foods and beverages consumed over the past 24 h, including timing, location, types and quantities of foods, and cooking methods. Nutritional analysis was conducted using the CANPRO 5.0 software developed by the Korean Nutrition Society. The nutritionists evaluated compliance and offered feedback using the Kini Care mobile application over the weekends. The Korean version of the MD-adherence questionnaire was employed to determine each patient's MD score (15). The Korean adaptation of the MD-adherence questionnaire was developed by our research team (15). It comprises 14 questions about dietary habits and the frequency of consumption of various foods, including perilla or olive oil, vegetables, fruit, red meat, butter and margarine, soft drinks, wine, beans and tofu, fish and seafood, sweets, nuts, poultry, and whole grains. Each component is scored 1 point if the specified criteria are met, for a maximum score of 14. The educational goal is to achieve a score of 10 or higher, indicating better adherence to the MD. To assess adherence, the questionnaire was administered by a nutritionist four times during the study (group 1 used as example): before the start of the experimental diet (1st assessment), after completion of the experimental diet (2nd assessment), after the washout period and before the start of the control diet (3rd assessment), and after completion of the control diet (4th assessment).

### 2.4 Clinical and biochemical analyses

Study visits were scheduled at four time points—at screening/baseline and after 4, 8, and 12 weeks. During these visits, height was measured with a precision of 0.1 cm, and body weight was measured using an electronic scale accurate to 0.01 kg. The body mass index (BMI) was subsequently calculated using the standard formula. The patient's systolic and diastolic blood pressures were measured in a seated position after at least 5 min of rest, using the right arm. Blood pressure was measured twice, with a 1-min interval between measurements, and the average of the two readings was recorded.

For blood analysis, samples were collected following a minimum fasting period of 8 h. Fasting glucose, total cholesterol, low-density lipoprotein cholesterol, non-high-density lipoprotein cholesterol, triglycerides, and high-density lipoprotein cholesterol were assessed using enzymatic color tests. The calcium level was determined via an absorbance assay, whereas phosphorus levels were measured using the Molybdate UV assay (Roche Cobas 8000 c702, Roche Diagnostics, Germany). Magnesium was measured using the colorimetric endpoint method. Serum 25-hydroxyvitamin D was assessed using an electrochemiluminescence binding assay, and parathyroid hormone levels were determined via an electrochemiluminescence immunoassay. Serum total protein and albumin levels were determined via a colorimetric assay (Roche Cobas 8000 c702).

Blood urea nitrogen was evaluated using a kinetic test involving urease and glutamate dehydrogenase, and creatinine levels were determined using an enzymatic method (Roche Cobas 8000 c702). Serum cystatin C was quantified using the particle-enhanced immunoturbidimetric assay (Roche Cobas 8000 c702).

The estimated glomerular filtration rate (eGFR) was calculated using the CKD Epidemiology Collaboration equation (16) and the Modification of Diet in Renal Disease equation (17). The eGFR was also computed based on cystatin C using a previously reported equation (18).

Serum sodium, potassium, and chloride levels were determined using the potentiometry method, whereas the total CO_2_ (tCO_2_) level was assessed with an absorbance assay. Human fibroblast growth factor 23 and adiponectin were quantified using enzyme-linked immunosorbent assays. Indoxyl sulfate was measured using the high-performance liquid chromatography-fluorescence method with the HPLC-1260/FLD instrument (SIGMA, USA).

For spot urine analysis, urinary sodium and potassium levels were measured using the potentiometry method, and urine protein and urine creatinine contents were evaluated using the turbidimetric method (Roche Cobas 8000 c702).

Blood was centrifuged for 10 min at 2,500 rpm, after which the plasma layer was transferred to separate vials and stored at −80°C until analysis. For analysis of cytokines, samples were de-identified and assigned a unique study number specific to the site and patient. Blinded sample analysis was performed using a digital immunoassay on a Luminex analyzer (Luminex, Austin, TX, USA). We used the Human Cytokine Base Kit A (R&D Systems, Inc., Minneapolis, MN, USA), a 12-plex assay.

### 2.5 Covariates and endpoints

Lifestyle factors such as smoking, alcohol consumption, and exercise were assessed at baseline by using self-reported questionnaires. Smoking status was dichotomized as current smokers and non-smokers. A patient was considered an alcohol user if alcohol was consumed one or more times a month. Physical activity was defined as engaging in moderate to vigorous exercise for at least 3 days a week.

The primary efficacy endpoint was a change in the serum potassium level. Secondary endpoints were changes in BMI, eGFR, and inflammatory cytokine level. Underlying diseases, such as diabetes and hypertension, as well as medication history, were also recorded using the questionnaires, and patients were categorized accordingly.

### 2.6 Statistical analysis

Data are presented as the means ± standard deviations for continuous variables and as frequencies (percentages) for categorical variables. This was a pilot study that required a sample of at least 12 patients per group for statistical testing. Considering the dropout rate, a total of 50 patients were included to ensure a sufficient sample size (19). An independent two-sample *t*-test was applied to compare the differences in baseline characteristics between the two groups. A paired *t*-test was conducted to assess the mean differences between the two groups. The differences between the conventional diet and MEDi-POB diet before and after both interventions were presented as the means of the estimated pre-post differences along with a 95% confidence interval. A linear mixed model was used to adjust for sequence and period. Two-sided *p*-values < 0.05 were considered statistically significant. All statistical analyses were performed using SAS version 9.4 (SAS Institute Inc., Cary, NC, USA) and R software (version 4.1.1; R Foundation for Statistical Computing, Vienna, Austria).

## 3 Results

### 3.1 Characteristics of study sample

Table 1 summarizes the baseline characteristics of the study sample. The following were similar between groups 1 and 2: age (66.3 ± 13.6 years vs. 65.9 ± 15.6 years, *p* = 0.931), sex (women: 32% vs. 56%, *p* = 0.087), and BMI (26.3 ± 3.5 kg/m^2^ vs. 27.4 ± 3.9 kg/m^2^, *p* = 0.976). The baseline eGFR values were comparable between groups 1 and 2 (CKD-Epidemiology Collaboration: 40.6 ± 12.0 vs. 39.6 ± 14.4, *p* = 0.780), as were the other blood chemistry concentrations. The prevalence of underlying conditions, such as diabetes and hypertension, did not differ between the two groups nor did smoking habits, alcohol consumption, and levels of physical activity.

**Table 1:** Baseline characteristics of study sample.

	**Group 1**	**Group 2**
Age, years	66.3 ± 13.6	65.9 ± 15.6
**Sex**, ***n*** **(%)**		
Men	17 (68%)	11 (44%)
Women	8 (32%)	14 (56%)
SBP, mmHg	139.8 ± 14.0	133.4 ± 11.4
DBP, mmHg	72.1 ± 12.5	66.9 ± 13.4
BMI, kg/m^2^	26.3 ± 3.5	27.4 ± 3.9
**Blood analysis**		
Total cholesterol, mg/dl	139.6 ± 28.5	153.8 ± 27.3
Triglycerides, mg/dl	131.8 ± 66.0	149.6 ± 71.9
HDL-C, mg/dl	48.3 ± 14.9	47.4 ± 12.7
LDL-C, mg/dl	73.8 ± 27.3	88.4 ± 24.7
Glucose, mg/dl	116.4 ± 38.5	123.4 ± 45.9
Calcium, mg/dl	9.3 ± 0.4	9.1 ± 0.4
Phosphate, mg/dl	3.6 ± 0.5	3.7 ± 0.5
Magnesium, mg/dl	2.11 ± 0.18	2.06 ± 0.21
25-hydroxyvitamin D, ng/ml	28.0 ± 15.2	27.1 ± 11.0
PTH, pg/ml	56.2 ± 41.3	61.8 ± 37.2
Total protein, g/dl	6.9 ± 0.5	6.8 ± 0.4
Albumin, g/dl	4.4 ± 0.4	4.3 ± 0.3
BUN, g/dl	28.4 ± 9.3	31.0 ± 12.1
Creatinine, g/dl	1.71 ± 0.47	1.74 ± 0.65
Cystatin C, mg/l	1.79 ±0.47	1.81 ± 0.44
eGFR (MDRD)	39.8 ± 11.1	38.9 ± 13.3
eGFR (CKD-EPI)	40.6 ± 12.0	39.6 ± 14.4
eGFR (cystatin C)	37.8 ±13.3	34.6 ± 14.8
Sodium, mmol/l	140.5 ± 2.3	140.7 ± 2.1
Potassium, mmol/l	4.81 ± 0.52	4.80 ± 0.42
Chloride, mmol/l	105.8 ± 2.5	105.4 ± 3.5
Total CO_2_, mmol/l	24.5 ± 2.9	24.6 ± 2.4
Human FGF23, pg/ml	15.1 ± 7.1	18.6 ±13.9
Adiponectin, ng/ml	15,973.4 ± 17,562.1	17,400.3 ± 18,850.9
Indoxyl sulfate, mg/dl	0.36 ± 0.31	0.33 ± 0.28
**Urine analysis**		
Protein, mg/dl	67.4 ± 86.8	40.6 ± 45.6
Creatinine, mg/dl	110.1 ± 56.4	84.0 ± 41.0
Protein/Creatinine ratio	612.0 ± 738.4	570.0 ± 618.5
Sodium, mmol/l	89.7 ± 33.5	84.1 ± 31.4
Potassium, mmol/l	43.1 ± 20.5	41.7 ± 23.6
Albumin, mg/dl	1.3 ± 1.3	0.9 ± 1.1
Current smoker, *n* (%)	0 (0%)	1 (4%)
Alcohol drinking, *n* (%)	8 (32%)	9 (36%)
Physically active, *n* (%)	5 (20%)	9 (36%)
**CKD stage**, ***n*** **(%)**		
Stage 3	20 (80%)	18 (72%)
Stage 4	5 (20%)	7 (28%)
Diabetes mellitus, *n* (%)	16 (64%)	16 (64%)
Hypertension, *n* (%)	21 (84%)	23 (92%)

Data are presented as means ± standard deviations, unless otherwise indicated.

SBP, systolic blood pressure; DBP, diastolic blood pressure; BMI, body mass index; HDL-C, high-density lipoprotein cholesterol; LDL-C, low-density lipoprotein cholesterol; PTH, parathyroid hormone; BUN, blood urea nitrogen; eGFR, estimated glomerular filtration rate; FGF23, fibroblast growth factor 23; CKD-EPI, CKD Epidemiology Collaboration equation; MDRD, Modification of Diet in Renal Disease equation; CKD, chronic kidney disease.

Group 1: patients initially followed the MEDi-POB diet and switched to the conventional diet.

Group 2: patients initially followed the conventional diet and switched to the MEDi-POB diet.

Table 2 presents the nutritional status of the study population. The following baseline characteristics were similar between groups 1 and 2: total caloric intake (1,178.4 ± 374.3 vs. 1,193.9 ± 539.4 kcal/day, *p* = 0.98), carbohydrate consumption (179.0 ± 62.0 vs. 171.2 ± 64.7 g/day, *p* = 0.67), protein intake (41.7 ± 16.7 vs. 50.7 ± 29.6 g/day, *p* = 0.19), fat consumption (31.5 ± 13.5 vs. 31.9 ± 29.4 g/day, *p* = 0.95), dietary fiber intake (15.5 ± 6.7 vs. 15.4 ± 8.2 g/day, *p* > 0.99), and potassium intake (1,576.9 ± 522.6 vs. 1,769.7 ± 856.2 mg/day, *p* = 0.35). The intakes of other nutritional components were also similar between the groups. However, the baseline MDS was significantly higher in group 1 compared to group 2 (5.7 ± 1.5 vs. 4.6 ± 1.7, respectively, *p* = 0.03). Specifically, group 1 was found to consume ≥3 servings of nuts per week more frequently (data not shown).

**Table 2:** Baseline nutritional status of the study sample.

	**Group 1**	**Group 2**	***p*-value**
MDS	5.7 ± 1.5	4.6 ± 1.7	0.03
Caloric intake, kcal	1,178.4 ± 374.3	1,193.9 ± 539.4	0.98
Carbohydrate, g	179.0 ± 62.0	171.2 ± 64.7	0.67
Fat, g	31.5 ± 13.5	31.9 ± 29.4	0.95
Protein, g	41.7 ± 16.7	50.7 ± 29.6	0.19
Carbohydrate, %	61.0 ± 8.6	62.7 ± 12.8	0.58
Fat, %	24.0 ± 6.8	21.4 ± 9.1	0.49
Protein, %	14.1 ± 3.3	14.9 ± 4.7	0.06
Fiber, g	15.5 ± 6.7	15.4 ± 8.2	>0.99
Vitamin A, μg RAE	253.4 ± 180.7	279.1 ± 277.7	0.70
Retinol, μg	72.1 ± 92.4	67.0 ± 108.6	0.86
β-carotene, μg	2,115.0 ± 2,048.7	2,554.4 ± 3,249.1	0.57
Vitamin E, mg	10.8 ± 6.5	9.8 ± 7.3	0.61
Vitamin K, μg	90.9 ± 138.3	151.1 ± 152.8	0.16
Vitamin C, mg	52.8 ± 49.7	63.5 ± 48.2	0.44
Thiamin, mg	1.09 ± 0.42	1.13 ± 0.75	0.82
Riboflavin, mg	0.87 ± 0.55	0.80 ± 0.52	0.67
Niacin, mg	7.0 ± 2.9	11.1 ± 11.3	0.09
Vitamin B12, μg	4.4 ± 4.9	8.7 ± 11.5	>0.99
Calcium, mg	288.3 ±148.4	247.7 ± 141.1	0.33
Phosphorus, mg	656.9 ± 268.9	659.4 ± 354.4	0.98
Sodium, mg	2,096.0 ± 1,395.6	2,534.8 ± 1,059.6	0.22
Potassium, mg	1,576.9 ± 522.6	1,769.7 ± 856.2	0.35
Magnesium, mg	76.3 ± 37.8	87.1 ± 48.2	0.39
Iron, mg	10.1 ± 4.3	12.0 ± 8.6	0.34
Zinc, mg	6.0 ± 2.1	6.2 ± 3.0	0.83
Copper, μg	496.8 ± 220.3	467.2 ± 315.0	0.71
Cholesterol, mg	222.1 ± 260.7	183.2 ± 231.2	0.58
Saturated fat, g	6.8 ± 3.7	6.9 ± 6.8	0.56
Monounsaturated fat, g	8.22 ± 4.74	8.19 ± 10.43	0.95
Polyunsaturated fat, g	9.53 ± 8.34	7.29 ± 7.06	0.32
N-3 PUFAs, g	0.56 ± 0.87	0.61 ± 1.09	0.86
N-6 PUFAs, g	3.97 ± 5.95	1.94 ± 2.06	0.13
N-3 PUFAs/N-6 PUFAs	0.15 ± 0.17	0.47 ± 0.10	0.13

Data are presented as daily means ± standard deviations.

MDS, Mediterranean diet score; RAE, retinol activity equivalents; PUFA, polyunsaturated fatty acid.

P-value was calculated using the independent t-test.

### 3.2 Anthropometric measurements and biochemical parameters

Table 3 presents the mean differences in anthropometric measurements and biochemical parameters after the MEDi-POB diet, after the control diet, and between the MEDi-POB and control diets. The order in which patients followed each diet did not influence the outcomes. A notable period effect was observed only in the mean changes in total cholesterol and chloride.

**Table 3:** Mean differences in anthropometric measurements and biochemical parameters based on the MEDi-POB and control diets.

	**Differences within the MEDi-POB diet**	**Differences within the control diet**	**Differences between the control diet and MEDi-POB diet**	***p1***	***p2***	***p3***
SBP, mmHg	1.57 (−2.87, 6.02)	1.03 (−3.41, 5.47)	−0.55 (−5.92, 4.83)	**-**	**-**	**-**
DBP, mmHg	1.26 (−1.98, 4.50)	1.40 (−1.84, 4.64)	0.14 (−4.12, 4.41)	**-**	**-**	**-**
BMI, kg/m^2^	−0.31 (−0.52, −0.11)	0.01 (−0.20, 0.21)	0.32 (0.04, 0.60)	**-**	**-**	^*^
**Blood analysis**				**-**	**-**	**-**
Total cholesterol, mg/dl	−5.00 (−10.00, 0.00)	−1.30 (−6.30, 3.70)	3.70 (−3.37, 10.77)	**-**	^*^	**-**
Triglycerides, mg/dl	−6.46 (−21.91, 8.99)	−3.51 (−18.96, 11.93)	2.95 (−18.57, 24.46)	**-**	**-**	**-**
HDL-C, mg/dl	−1.06 (−2.93, 0.82)	−0.81 (−2.68, 1.06)	0.25 (−2.40, 2.90)	**-**	**-**	**-**
LDL-C, mg/dl	−0.41 (−4.09, 3.28)	−0.15 (−3.83, 3.54)	0.26 (−4.95, 5.47)	**-**	**-**	**-**
Glucose, mg/dl	0.44 (−13.51, 14.39)	−3.10 (−17.05, 10.85)	−3.54 (−20.60, 13.52)	**-**	**-**	**-**
Calcium, mg/dl	0.003 (−0.099, 0.105)	0.010 (−0.092, 0.112)	0.007 (−0.137, 0.151)	**-**	**-**	**-**
Phosphate, mg/dl	−0.03 (−0.18, 0.13)	0.10 (−0.06, 0.26)	0.13 (−0.09, 0.35)	**-**	**-**	**-**
Magnesium, mg/dl	0.03 (−0.02, 0.08)	0.00 (−0.05, 0.05)	−0.03 (−0.09, 0.03)	**-**	**-**	**-**
25-hydroxyvitamin D, ng/ml	1.00 (−0.36, 2.35)	0.07 (−1.30, 1.43)	−0.93 (−2.86, 1.00)	**-**	**-**	**-**
PTH, pg/ml	0.78 (−5.02, 6.57)	−2.22 (−8.02, 3.57)	−3.00 (−10.74, 4.74)	**-**	**-**	**-**
Total protein, g/dl	0.09 (−0.00, 0.19)	0.10 (0.00, 0.19)	0.00 (−0.13, 0.14)	**-**	**-**	**-**
Albumin, g/dl	0.01 (−0.05, 0.07)	−0.01 (−0.07, 0.05)	−0.03 (−0.11, 0.06)	**-**	**-**	**-**
BUN, g/dl	−0.71 (−2.97, 1.55)	0.76 (−1.50, 3.02)	1.47 (−1.73, 4.67)	**-**	**-**	**-**
Creatinine, g/dl	0.01 (−0.06, 0.07)	0.09 (0.02, 0.15)	0.08 (−0.00, 0.16)	**-**	**-**	**-**
Cystatin C, mg/l	0.01 (−0.04, 0.07)	0.06 (0.01, 0.12)	0.05 (−0.02, 0.12)	**-**	**-**	**-**
eGFR (MDRD)	0.57 (−0.99, 2.14)	−2.35 (−3.92, −0.79)	−2.93 (−5.14, −0.72)	**-**	**-**	^*^
eGFR (CKD-EPI)	0.62 (−1.09, 2.33)	−2.57 (−4.27, −0.86)	−3.19 (−5.60, −0.78)	**-**	**-**	^*^
eGFR (cystatin C)	−0.37 (−1.61, 0.86)	−1.47 (−2.73, −0.21)	−1.10 (−2.87, 0.67)	**-**	**-**	**-**
Sodium, mmol/l	−0.59 (−1.24, 0.06)	−0.49 (−1.14, 0.16)	0.10 (−0.82, 1.01)	**-**	**-**	**-**
Potassium, mmol/l	−0.025 (−0.165, 0.115)	−0.01 (−0.15, 0.13)	0.01 (−0.19, 0.21)	**-**	**-**	**-**
Chloride, mmol/l	−1.19 (−1.93, −0.46)	−0.58 (−1.31, 0.16)	0.61 (−0.41, 1.64)	**-**	^*^	**-**
Total CO_2_, mmol/l	1.01 (0.05, 1.97)	−0.33 (−1.29, 0.63)	−1.35 (−2.64, −0.05)	**-**	**-**	^*^
Human FGF23, pg/ml	−0.12 (−2.72, 2.48)	2.90 (0.30, 5.50)	3.02 (−0.65, 6.69)	**-**	**-**	**-**
Adiponectin, ng/ml	2,406.5 (−1,414.3, 6,227.2)	−414.5 (−4,235.3, 3,406.3)	−2,821.0 (−8,224.4, 2,582.4)	**-**	**-**	**-**
Indoxyl sulfate, mg/dl	−0.03 (−0.10, 0.04)	0.06 (−0.01, 0.13)	0.09 (−0.01, 0.18)	**-**	**-**	**-**
**Urine analysis**				**-**	**-**	**-**
Protein, mg/dl	0.27 (−12.80, 13.34)	−6.75 (−19.82, 6.32)	−7.02 (−25.50, 11.46)	**-**	**-**	**-**
Creatinine, mg/dl	9.32 (−6.85, 25.50)	2.11 (−14.07, 18.28)	−7.22 (−30.09, 15.66)	**-**	**-**	**-**
Protein/Creatinine ratio	−62.52 (−198.20, 73.16)	−11.73 (−147.41, 123.96)	50.80 (−141.09, 242.68)	**-**	**-**	**-**
Sodium, mmol/l	−5.07 (−16.51, 6.38)	−1.53 (−13.04, 9.99)	3.54 (−11.69, 18.78)	**-**	**-**	**-**
Potassium, mmol/l	2.21 (−3.62, 8.04)	−4.98 (−10.85, 0.88)	−7.19 (−15.46, 1.08)	**-**	**-**	**-**
Albumin, mg/dl	−0.15 (−0.34, 0.04)	−0.07 (−0.26, 0.12)	0.08 (−0.14, 0.31)	**-**	**-**	**-**

Data are presented as mean differences (95% confidence intervals).

MEDi-POB, Mediterranean Proper Optimal Balance; SBP, systolic blood pressure; DBP, diastolic blood pressure; BMI, body mass index; HDL-C, high-density lipoprotein cholesterol; LDL-C, low-density lipoprotein cholesterol; PTH, parathyroid hormone; BUN, blood urea nitrogen; eGFR, estimated glomerular filtration rate; FGF23, fibroblast growth factor 23; Cr, creatinine; CKD-EPI, CKD epidemiology collaboration equation; MDRD, Modification of Diet in Renal Disease equation; CKD, chronic kidney disease.

*p*1, p-value for difference between sequences; *p*2, p-value for difference between periods; *p*3, p-value for difference between diets.

^*^p-value < 0.05.

After adjusting for sequence and period carry-over effects, the BMI slightly decreased from baseline and differed between the MEDi-POB and control diets (*p* for difference between diets = 0.028). Furthermore, the change in eGFR following the MEDi-POB diet was not significant (*p* = 0.287) while the control diet yielded a slight decrease in eGFR (*p* = 0.010). Consequently, the post-intervention eGFR (both Modification of Diet in Renal Disease and CKD-Epidemiology Collaboration) was higher following the MEDi-POB diet than following the control diet (*p*-value for difference between diets = 0.011). Additionally, altough the MEDi-POB diet yielded a slight increase in tCO_2_ compared to the control diet, leading to higher post-intervention tCO_2_ levels following the MEDi-POB diet (*p*-value for difference between diets = 0.043). The comparisons of anthropometric measurements and biochemical parameters before and after each intervention is summarized in Supplementary Table 3.

Table 4 displays mean differences in nutritional status after the MEDi-POB diet, after the control diet, and between the MEDi-POB and control diets. A significant sequence effect was observed in the mean differences in the MDS and dietary niacin intake. Additionally, a noteworthy period effect emerged in the mean differences in protein intake proportion and dietary magnesium and copper intakes.

**Table 4:** Mean differences in daily nutritional status according to the diet.

	**Differences within the MEDi-POB diet**	**Differences within the control diet**	**Differences between the control diet and MEDi-POB diet**	***p1***	***p2***	***p3***
MDS	4.93 (4.43, 5.44)	−0.21 (−0.71, 0.29)	−5.14 (−5.81, −4.48)	^*^	**-**	^*^
Caloric intake, kcal	222.96 (55.81, 390.10)	−103.79 (−270.93, 63.36)	−326.74 (−563.12, −90.36)	**-**	**-**	^*^
Carbohydrate, g	19.12 (−3.46, 41.69)	−12.30 (−34.87, 10.27)	−31.42 (−63.34, 0.51)	**-**	**-**	**-**
Fat, g	17.60 (8.18, 27.02)	−5.06 (−14.48, 4.36)	−22.67 (−35.99, −9.35)	**-**	**-**	^*^
Protein, g	4.32 (−4.16, 12.80)	−0.93 (−9.41, 7.54)	−5.25 (−17.24, 6.74)	**-**	**-**	**-**
Carbohydrate, %	−3.26 (−7.66, 1.14)	−0.19 (−4.59, 4.21)	3.07 (−3.15, 9.29)	**-**	**-**	**-**
Fat, %	6.68 (3.26, 10.1)	−0.00 (−3.41, 3.41)	– 6.68 (−11.50, −1.85)	**-**	**-**	^*^
Protein, %	−0.98 (−2.77, 0.81)	0.40 (−1.39, 2.19)	1.38 (−1.05, 3.81)	**-**	^*^	**-**
Fiber, g	5.99 (3.58, 8.40)	−1.03 (−3.44, 1.38)	−7.02 (−10.43, −3.61)	**-**	**-**	^*^
Vitamin A, μg RAE	14.67 (−75.29, 104.64)	24.40 (−65.57, 114.37)	9.73 (−102.84, 122.30)	**-**	**-**	**-**
Retinol, μg	−43.93 (−77.35, −10.52)	3.22 (−30.20, 36.64)	47.15 (−0.11, 94.41)	**-**	**-**	**-**
β-carotene, μg	−341.27 (−1,342.58, 659.99)	284.71 (−716.55, 1,285.96)	625.98 (−624.53, 1,876.49)	**-**	**-**	**-**
Vitamin E, mg	−0.27 (−2.48, 1.93)	−0.13 (−2.34, 2.07)	0.14 (−2.61, 2.89)	**-**	**-**	**-**
Vitamin K, μg	−15.20 (−76.39, 46.0)	−7.76 (−68.96, 53.43)	7.43 (−75.61, 90.48)	**-**	**-**	**-**
Vitamin C, mg	29.13 (8.91, 49.35)	6.02 (−14.20,26.24)	−23.11 (−51.01, 4.78)	**-**	**-**	**-**
Thiamin, mg	−0.15 (−0.37, 0.08)	−0.06 (−0.28, 0.16)	0.09 (−0.22, 0.39)	**-**	**-**	**-**
Riboflavin, mg	0.06 (−0.12, 0.24)	0.08 (−0.10, 0.26)	0.02 (−0.21, 0.25)	**-**	**-**	**-**
Niacin, mg	3.36 (1.23, 5.50)	−0.89 (−3.02, 1.25)	−4.25 (−7.27, −1.23)	^*^	**-**	^*^
Vitamin B12, μg	−2.97 (−5.88, −0.05)	−1.29 (−4.20, 1.63)	1.68 (−2.44, 5.80)	**-**	**-**	**-**
Calcium, mg	7.58 (−50.51, 65.70)	16.0 (−42.09, 74.09)	8.42 (−73.73, 90.57)	**-**	**-**	**-**
Phosphorus, mg	−14.44 (−114.80, 85.93)	−4.31 (−104.68, 96.06)	10.12 (−131.82,152.06)	**-**	**-**	**-**
Sodium, mg	−761.21 (−1154.14, −368.28)	−150.63 (−543.56, 242.30)	610.57 (54.89, 1166.26)	**-**	**-**	^*^
Potassium, mg	94.23 (−92.22, 280.67)	−166.18 (−352.63, 20.27)	−260.41 (−524.08, 3.27)	**–**	**-**	**-**
Magnesium, mg	−14.95 (−26.43,−3.46)	−14.36 (−25.85, −2.87)	0.59 (−15.33, 16.51)	**-**	^*^	**-**
Iron, mg	−2.29 (−4.45, −0.14)	−0.73 (−2.89, 1.42)	1.56 (−1.33, 4.45)	**-**	**-**	**-**
Zinc, mg	−1.02 (−2.31, 0.28)	0.42 (−0.87, 1.71)	1.44 (−0.19, 3.07)	**-**	**-**	**-**
Copper, μg	−145.10 (−220.40, −71.59)	−33.59 (−107.99, 40.82)	112.41 (7.18, 217.64)	**-**	^*^	^*^
Cholesterol, mg	−53.33 (−124.10, 17.43)	40.34 (−30.43, 111.11)	93.68 (−6.41, 193.76)	**-**	**-**	**-**
Saturated fat, g	1.15 (−0.83, 3.13)	−0.10 (−2.08, 1.88)	−1.25 (−4.05, 1.55)	**-**	**-**	^*^
Monounsaturated fat, g	6.69 (3.86, 9.52)	0.13 (−2.70, 2.96)	−6.56 (−10.56, −2.56)	**-**	**-**	^*^
Polyunsaturated fat, g	4.39 (2.07, 6.70)	−0.57 (−2.89, 1.74)	−4.96 (−8.23, −1.68)	**-**	**-**	^*^
N-3 PUFAs, g	0.78 (0.46, 1.11)	−0.26 (−0.58, 0.07)	−1.04 (−1.47, −0.61)	**-**	**-**	^*^
N-6 PUFAs, g	5.26 (3.66, 6.86)	−0.19 (−1.79, 1.41)	−5.45 (−7.71, −3.19)	**-**	**-**	^*^
N-3 PUFAs/N-6 PUFAs	−0.05 (−0.22, 0.13)	−0.18 (−0.35, 0.26)	−0.13 (−0.34, 0.07)	**-**	**-**	**-**

MEDi-POB, Mediterranean Proper Optimal Balance; MDS, Mediterranean diet score; PUFA, poly unsaturated fatty acid.

*p*1, p-value for difference between sequences; *p*2, p-value for difference between periods; *p*3, p-value for difference between diets.

^*^p-value < 0.05.

After adjusting for sequence and period carry-over effects, the MDS significantly increased in the MEDi-POB diet group (by 4.93 points; *p* < 0.001), while it slightly decreased, though not significantly, in the control diet group (−0.21 points). Overall, the MEDi-POB diet substantially improved MDS compared to the control diet (*p* for difference between diets < 0.001). This improvement was reflected in significant enhancements in adherence to critical dietary practices, including the use of perilla or olive oil as the principal cooking fat, as well as increased consumption of perilla or olive oil, vegetables, fish or seafood, nuts, and whole grains. Additionally, there was a significant reduction in the consumption of red meat and sweets within the MEDi-POB diet group, indicating a shift toward healthier dietary habits in alignment with the Mediterranean diet framework (Supplementary Table 4).

Moreover, significant increases were observed in total caloric intake (*p* for difference between diets = 0.008), as well as in the intake of monounsaturated (*p* for difference between diets = 0.002), polyunsaturated (*p* for difference between diets = 0.004), omega-3 (*p* for difference between diets < 0.001), and omega-6 (*p* for difference between diets < 0.001) fatty acids, dietary fiber (*p* for difference between diets = 0.001), and niacin (*p* for difference between diets = 0.007) following the MEDi-POB diet compared to the control diet. Specifically, the total caloric intake increased by 222.96 kcal/day (*p* = 0.013) after the MEDi-POB diet, whereas it decreased by 103.79 kcal/day (*p* = 0.221) following the control diet. Conversely, significant decreases were noted in sodium and copper intakes following the MEDi-POB diet compared to those following the control diet. An increase in dietary potassium intake was observed after the MEDi-POB diet (94.2 mg/day; *p* = 0.263), whereas a decrease was observed after the control diet (−166.2 mg/day; *p* = 0.072); however, the difference between the diets was not significant (*p* for difference between diets = 0.053). The comparison of nutritional status before and after each diet is summarized in Supplementary Table 5.

**Figure 2:**
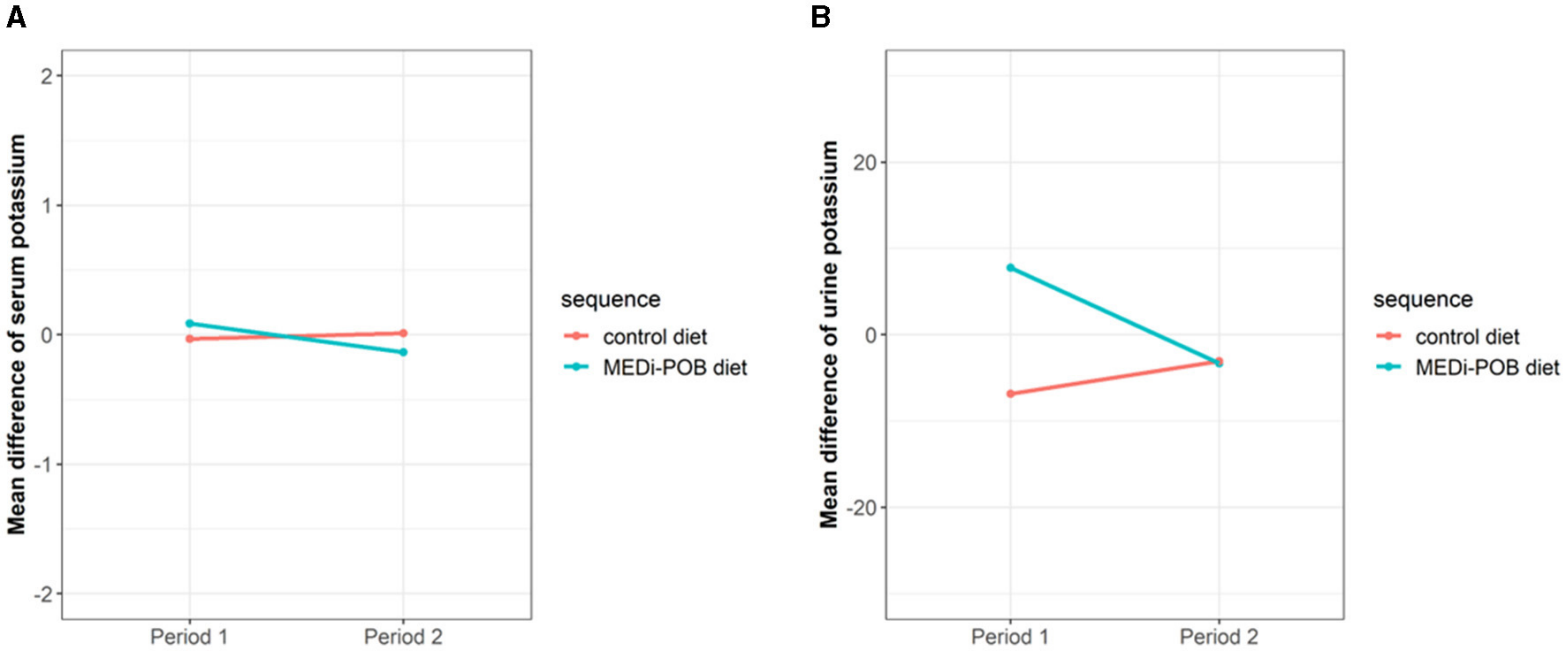
Mean difference plot of serum and urine potassium levels after intervention during periods 1 and 2. **(A)** Serum potassium levels. **(B)** Urine potassium levels.

Figure 2 illustrates a mean difference plot depicting the changes in serum (Figure 2A) and urine (Figure 2B) potassium levels after the interventions during periods 1 and 2. During both periods, no significant changes were observed in the serum or urine potassium levels after either diet.

### 3.3 Differences in cytokines between MEDi-POB and control diets

Supplementary Table 6 summarizes the baseline cytokine levels of the study sample. No significant differences in serum cytokine levels were observed between groups 1 and 2. Table 5 displays the mean differences in serum cytokine levels after the MEDi-POB diet, after the control diet, and between the two diets. Both a significant sequence effect and a significant period effect were observed in the mean differences in the cytokine interleukin 2 level. After adjusting for sequence and period carry-over effects, a significant decrease was discovered in granulocyte-macrophage colony-stimulating factor after the MEDi-POB diet compared to that after the control diet.

**Table 5:** Mean changes in cytokines according to the MEDi-POB and control diets.

**Cytokines**	**Differences within the MEDi-POB diet**	**Differences within the control diet**	**Differences between the control diet and MEDi-POB diet**	***p1***	***p2***	***p3***
GM-CSF	−1.55 (−2.94, −0.16)	−0.30 (−1.70, 1.11)	1.25 (−0.72, 3.23)	**-**	**-**	^*^
INF-r	3.97 (−1.69, 9.63)	−1.64 (−7.36, 4.08)	−5.61 (−13.65, 2.44)	**-**	^*^	**-**
IL-1β	−3.84 (−7.76, 0.08)	0.31 (−3.65, 4.27)	4.16 (−1.41, 9.73)	**-**	**-**	**-**
IL-10	0.35 (−0.68, 1.38)	−0.37 (−1.41, 0.68)	−0.72 (−2.17, 0.73)	**-**	**-**	**-**
IL-12p70	−1.90 (−11.42, 7.62)	−7.79 (−17.40, 1.82)	−5.88 (−17.48, 5.71)	**-**	**-**	**-**
IL-2	0.18 (−1.28, 1.65)	−0.88 (−2.36, 0.60)	−1.06 (−3.03, 0.90)	^*^	^*^	
IL-4	−7.46 (−19.88, 4.97)	−6.15 (−18.68, 6.38)	1.31 (−11.91, 14.52)	**-**	**-**	**-**
IL-5	0.17 (−0.35, 0.68)	−0.18 (−0.70, 0.34)	−0.35 (−1.08, 0.38)	**-**	**-**	**-**
IL-6	0.31 (−2.54, 3.15)	1.93 (−0.95, 4.81)	1.63 (−2.31, 5.55)	**-**	**-**	**-**
IL-8	−86.90 (−294.80, 121.00)	115.66 (−94.39, 325.72)	202.57 (−92.98, 498.11)	**-**	**-**	**-**
TNF-α	−1.07 (−4.83, 2.70)	−0.03 (−3.83, 3.78)	1.04 (−4.31, 6.39)	**-**	**-**	**-**
VEGF	−5.00 (−23.26, 13.26)	10.70 (−7.75, 29.14)	15.70 (−10.26, 41.65)	**-**	**-**	**-**

MEDi-POB, Mediterranean Proper Optimal Balance; GM-CSF, granulocyte-macrophage colony-stimulating factor; INF, interferon; IL, interleukin; TNF-α, tumor necrosis factor alpha; VEGF, vascular endothelial growth factor.

*p*1, p-value for difference between sequences; *p*2, p-value for difference between periods; *p*3, p-value for difference between groups.

^*^Asterix means that the p-value is < 0.05.

## 4 Discussion

In this randomized, controlled, crossover trial including patients with stage 3–4 CKD, dietary potassium intake did not significantly differ between the diets, but it increased in the MEDi-POB group. Nonetheless, no significant changes were observed in serum or urine potassium levels following the MEDi-POB diet. Kidney function remained well-preserved following the MEDi-POB diet. We observed that dietary fat, fiber, and niacin intakes increased following the MEDi-POB diet compared to those following the control diet, whereas sodium and copper intakes significantly decreased. The MEDi-POB diet led to an increased caloric intake but a slightly decreased BMI compared to the control diet.

As CKD progresses, individuals face a spectrum of nutritional challenges, including undernutrition, protein-energy wasting, micronutrient deficiencies, and electrolyte imbalances, while paradoxically, obesity becomes more prevalent across all CKD stages (20). Most of our study patients, although obese, also had insufficient intake of calories and several micronutrients and excessive consumption of carbohydrates and sodium.

Although high-potassium diets are generally advised for the general population, individuals with CKD often have to restrict their dietary potassium intake owing to concerns about hyperkalemia (8, 9); however, these restrictions, supported by limited empirical evidence, can inadvertently lead to reduced consumption of nutritious foods in such individuals (21). In two recent population-based studies, individuals with CKD consumed fewer fruits and vegetables compared to those without the disease, and a decreased frequency of potassium-rich food intake was significantly associated with an increased risk of mortality, regardless of CKD status (22, 23). In contrast, a greater adherence to the MD independently predicted survival in patients with CKD (7). The MD promotes the consumption of potassium-rich alkaline foods, including fruits, vegetables, legumes, whole grains, and nuts. In addition, this diet is characterized by a predominance of vegetal protein, intake of monounsaturated fats and polyunsaturated fatty acids over saturated fats, high dietary fiber intake, and low food-derived oxidative stress and inflammation (24). Prior studies have consistently demonstrated that the MD effectively preserves kidney function and slows the progression of CKD (6, 25, 26).

Our study, revealed no significant changes in serum or urine potassium levels with the MEDi-POB diet, consistent with previous research findings. The exact relationship between the MD and hyperkalemia is not fully understood. However, the results of previous studies suggest that it may be due to the lower bioavailability of potassium in unprocessed and minimally processed foods (27). Additionally, intact plant cell walls may mitigate the effect on serum potassium levels, and the base-inducing nature of a plant-based diet may shift potassium intracellularly (28). Nonetheless, further research is needed, particularly in patients with advanced CKD.

The MD is well-known to provide additional benefits, including weight reduction, blood pressure control, and control of the low-density lipoprotein cholesterol level (29). Reflecting prior research, the MEDi-POB diet led to a noteworthy increase in caloric intake and a slight reduction in BMI compared to the control diet. Moreover, the MEDi-POB diet significantly improved overall MDS and adherence to key Mediterranean dietary practices, promoting healthier eating habits than the control diet. It also increases the intake of various dietary components, including fat, fiber, and niacin, which aligns with studies indicating the potential of polyunsaturated fatty acids to alleviate proteinuria, lower triglycerides, and reduce inflammation in CKD (30). A crucial point to emphasize is that a low-fiber diet can boost the production of uremic molecules owing to increased intestinal proteolytic activity. On a positive note, extensive observational studies have demonstrated a 40–50% reduction in CKD risk with a high fiber intake (31). Furthermore, increasing niacin intake may help lower oxidative stress and phosphate levels (32). Notably, the MEDi-POB diet yielded a slight increase in tCO_2_, often used as a surrogate for acid-base parameters in CKD, signifying effective management of metabolic acidosis in patients with CKD (33). Conversely, the MEDi-POB diet significantly reduced sodium and copper intake. Meta-analyses of randomized controlled trials support moderate salt restriction for reducing blood pressure and proteinuria, and a low-salt diet may decrease kidney composite outcomes in patients with CKD (34). Excessive dietary copper intake can result in copper deposition in the kidneys, leading to nephrotoxicity characterized by proximal tubule necrosis due to oxidative stress and cellular damage, ultimately reducing kidney function (35). These favorable changes resulting from the MEDi-POB diet contributed to preserved kidney function, as evidenced by the lack of significant alterations from baseline in the potassium, parathyroid hormone, and fibroblast growth factor 23 levels, as well as reduction in pro-inflammatory cytokines such as granulocyte-macrophage colony-stimulating factor (36, 37).

Our study has some limitations. First, the 4-week intervention duration may not have been sufficient to observe significant changes in metabolic parameters or conclusively assess CKD prognosis. This limitation warrants extensive and long-term investigations to comprehensively evaluate the potential impacts of the MD on metabolic parameters and CKD prognosis. Second, despite receiving the same caloric education and guidance according to Kidney Disease Outcomes Quality Initiative guidelines, neither group met the recommended caloric intake. This is likely because, at baseline, patients in both groups consumed fewer calories than the recommended quantity, aligning with recent studies indicating that many older Korean adults and patients with CKD often suffer from nutritional deficiencies (38, 39). However, the MEDi-POB diet was associated with an increased caloric intake, whereas the control diet was associated with a non-significant decrease. Third, nutrient intake was assessed using the 24-h dietary recall method in this study, which might have introduced recall bias. Finally, this was a pilot study conducted to explore the feasibility of the research, and therefore, conclusive interpretations cannot be made from these results. However, for the primary endpoint of serum potassium level, a *post-hoc* calculation revealed a power of 87.7% in the current study.

Nevertheless, our study has several strengths. It was a randomized, crossover-controlled trial with a control group. The MEDi-POB diet comprised tailored home-delivered meals following Mediterranean principles, ensuring accurate dietary intervention. We used the validated Korean version of the MD-adherence questionnaire to assess adherence to the MD. We minimized recall bias by monitoring food intake on a daily basis and assessing compliance via a mobile application throughout the intervention. Accordingly, the study achieved exceptional compliance rates, exceeding 90%.

## 5 Conclusion

In conclusion, the MEDi-POB diet resulted in a non-significant increase in dietary potassium intake without significant changes in serum and urine potassium levels. Kidney function remained well-preserved throughout the study. These results suggest that the MEDi-POB diet is safe even in patients with advanced CKD, as it does not adversely impact serum and urine potassium levels and helps maintain kidney function. Future research should be conducted to explore the long-term effects of the MEDi-POB diet on kidney health and its potential benefits for the management of CKD-related complications.

## Data Availability

The raw data supporting the conclusions of this article will be made available by the authors, without undue reservation.
