# Supplementary material for: Safety and impact of the Mediterranean diet in patients with chronic kidney disease: a pilot randomized crossover trial
Source: Front Nutr. 2024 Sep 4;11:1463502. doi: 10.3389/fnut.2024.1463502 (PMC11408278; doi:10.3389/fnut.2024.1463502)
Supplement: Supplementary file 1 [file Table_1.docx]

Supplementary Material

**Supplementary Table 1. The MEDI-POB diet menu and nutritional composition used in the study**

| Menus | Total calorie, kcal | Protein, g | Sodium, mg | Potassium, mg | Fiber, g |
| --- | --- | --- | --- | --- | --- |
| Chicken breast cheese rice bowl | 425 | 21 | 250 | 583.9 | 2.4 |
| Chicken breast nutrition rice bowl | 435 | 22.8 | 449.3 | 687.8 | 4.1 |
| Spicy grilled chicken rice bowl | 457 | 17.9 | 777.4 | 596.1 | 2.9 |
| Beef and seafood rice bowl | 457 | 19.1 | 402.2 | 594.8 | 3.2 |
| Alio e Olio Rice | 421 | 17 | 573.5 | 694.2 | 4.3 |
| Tofu and shrimp rice bowl | 455 | 16 | 357.1 | 644.9 | 5.6 |
| Tofu and pork rice bowl | 487 | 17.3 | 409.9 | 642.9 | 5.5 |
| Salmon steak | 450 | 21.2 | 445.7 | 730.4 | 4.1 |
| Squid and pork rice bowl | 445 | 19 | 380 | 578.7 | 4.3 |
| King-sized pork roast and burdock rice bowl | 517 | 19.2 | 496.9 | 583.4 | 3.1 |
| Standard liquid nutritional supplement Product name: Newcare KD  (200ml/1can) | 400 | 6 | 120 | 150 | 4 |
| Apple (1/2 piece) 100g | 49 | 0.3 | 4 | 110 | 1.5 |
| Product name: Fiber easy | 20 |  |  |  | 8 |
| Whole wheat bread (30g) | 78 | 2.5 | 181 | 50 | 1.7 |
| 1 teaspoon of olive oil (1teaspoon) | 46 | 0 | 0.15 | 0.2 | 0.5 |

**Supplementary Table 2. The example meal plans based on the MEDi-POB diet**

|  | 1day | 2day | 3day | 4day | 5day | 6day | 7day |
| --- | --- | --- | --- | --- | --- | --- | --- |
| Lunch | Chicken breast nutrition rice bowl | Alio e Olio Rice | Chicken breast cheese rice bowl | Chicken breast nutrition rice bowl | Tofu and shrimp rice bowl | Alio e Olio Rice | Beef and seafood rice bowl |
| Snacks | Newcare KD  (100ml) | Newcare KD  (100ml) | Newcare KD  (100ml) | Newcare KD  (100ml) | Newcare KD  (100ml) | Newcare KD  (100ml) | Newcare KD  (100ml) |
| Dinner | King-sized pork roast and burdock rice bowl | Salmon steak | Squid and pork rice bowl | Beef and seafood rice bowl | Spicy grilled chicken rice bowl | Tofu and pork rice bowl | Squid and pork rice bowl |
| Snacks |  | Apple (1/2 piece) 100g | Apple (1/2 piece) 100g | Apple (1/2 piece) 100g |  |  |  |

**Supplementary Table 3. Anthropometric measurements and biochemical parameters at baseline and after intervention based on the MEDi-POB diet and control diet.**

|  | Medi-POB diet | |  | Control diet | |  |
| --- | --- | --- | --- | --- | --- | --- |
|  | Pre | Post | P-value | Pre | Post | P-value |
| SBP, mmHg | 135.3 ± 13.6 | 136.1 ± 17.4 | 0.494 | 135.5 ± 13.2 | 136.7 ± 17.4 | 0.591 |
| DBP, mmHg | 68.9 ± 13.8 | 70.6 ± 12.6 | 0.451 | 68.5 ± 13.3 | 70.0 ± 14.4 | 0.318 |
| BMI, kg/m^2^ | 26.7 ± 3.7 | 26.6 ± 3.5 | 0.016 | 26.9 ± 3.7 | 26.8 ± 3.7 | 0.976 |
| Blood analysis |  |  |  |  |  |  |
| Total cholesterol, mg/dl | 145.0 ± 29.3 | 140.7 ± 36.9 | 0.092 | 147.7 ± 29.9 | 146.0 ± 27.8 | 0.461 |
| Triglyceride, mg/dl | 141.0 ± 67.0 | 136.6 ± 60.9 | 0.359 | 138.2 ± 70.7 | 134.3 ± 60.2 | 0.606 |
| HDL-C, mg/dl | 47.5 ± 13.8 | 46.1 ± 13.4 | 0.417 | 47.7 ± 13.6 | 46.9 ± 13.8 | 0.340 |
| LDL-C, mg/dl | 81.0 ± 28.5 | 81.4 ± 29.5 | 0.774 | 84.4 ± 27.2 | 84.1 ± 25.8 | 0.855 |
| Glucose, mg/dl | 119.9 ± 40.0 | 120.1 ± 45.8 | 0.989 | 116.4 ± 40.1 | 113.1 ± 30.8 | 0.624 |
| Calcium, mg/dl | 9.2 ± 0.4 | 9.2 ± 0.4 | 0.913 | 9.1 ± 0.4 | 9.1 ± 0.4 | 0.867 |
| Phosphate, mg/dl | 3.7 ± 0.6 | 3.6 ± 0.6 | 0.674 | 3.5 ± 0.5 | 3.6 ± 0.5 | 0.202 |
| Magnesium, mg/dl | 2.08 ± 0.18 | 2.10 ± 0.22 | 0.271 | 2.06 ± 0.18 | 2.06 ± 0.21 | 0.984 |
| 25-hydroxyvitamin D, ng/ml | 27.5 ± 13.4 | 29.2 ± 11.8 | 0.116 | 28.4 ± 12.4 | 28.9 ± 12.9 | 0.936 |
| PTH, pg/ml | 56.3 ± 36.4 | 58.8 ± 34.9 | 0.774 | 61.5 ± 38.9 | 59.1 ± 35.5 | 0.506 |
| Total protein, g/dl | 6.8 ± 0.4 | 6.9 ± 0.4 | 0.087 | 6.8 ± 0.4 | 6.9 ± 0.4 | 0.029 |
| Albumin, g/dl | 4.3 ± 0.3 | 4.3 ± 0.3 | 0.599 | 4.3 ± 0.3 | 4.3 ± 0.3 | 0.476 |
| BUN, g/dl | 29.0 ± 9.7 | 28.9 ± 11.6 | 0.593 | 29.3 ± 10.7 | 29.9 ± 9.3 | 0.557 |
| Creatinine, g/dl | 1.77 ± 0.56 | 1.81 ± 0.62 | 0.911 | 1.75 ± 0.59 | 1.83 ± 0.60 | 0.008 |
| Cystatin C, mg/l | 1.86 ± 0.49 | 1.89 ± 0.53 | 0.519 | 1.78 ± 0.45 | 1.87 ± 0.49 | 0.036 |
| eGFR (MDRD) | 38.0 ± 12.1 | 38.2 ± 13.1 | 0.292 | 39.4 ± 12.9 | 37.0 ± 12.0 | 0.009 |
| eGFR (CKD-EPI) | 38.7 ± 12.9 | 38.8 ± 14.1 | 0.287 | 40.0 ± 13.7 | 37.4 ± 12.6 | 0.010 |
| eGFR (cystatin C) | 36.1 ± 13.9 | 35.7 ± 13.7 | 0.580 | 37.6 ± 12.7 | 35.7 ± 13.5 | 0.036 |
| Sodium, mmol/l | 140.3 ± 2.0 | 139.8 ± 2.3 | 0.134 | 140.5 ± 2.2 | 140.0 ± 1.82 | 0.116 |
| Potassium, mmol/l | 4.81 ± 0.49 | 4.80 ± 0.51 | 0.634 | 4.87 ± 0.44 | 4.85 ± 0.50 | 0.845 |
| Chloride, mmol/l | 105.3 ± 3.0 | 104.2 ± 3.3 | 0.004 | 105.4 ± 3.2 | 104.8 ± 2.4 | 0.104 |
| Total CO_2_, mmol/l | 24.2 ± 4.2 | 25.1 ± 2.5 | 0.082 | 24.7 ± 2.5 | 24.3 ± 2.7 | 0.349 |
| Human FGF2, pg/ml | 19.5 ± 17.9 | 19.7 ± 14.9 | 0.802 | 16.8 ± 11.9 | 19.8 ± 13.4 | 0.027 |
| Adiponectin, ng/ml | 19375.6 ± 23957.6 | 21667.3 ± 34002.0 | 0.279 | 19447.4 ± 30764.0 | 19072.2±26520.5 | 0.785 |
| Indoxyl sulfate, mg/dl | 0.33 ± 0.27 | 0.31± 0.21 | 0.447 | 0.36 ± 0.29 | 0.42 ± 0.37 | 0.127 |
| Urine analysis |  |  |  |  |  |  |
| Creatinine, mg/dl | 56.1± 72.6 | 53.7 ± 80.4 | 0.959 | 58.5 ± 94.5 | 52.4 ± 78.8 | 0.331 |
| Protein/Creatinine ratio | 103.7 ± 51.8 | 113.0 ± 55.3 | 0.241 | 99.5 ± 49.5 | 101.8 ± 59.1 | 0.790 |
| Sodium, mmol/l | 568.3 ± 671.0 | 511.3 ± 658.0 | 0.487 | 614.7 ± 715.9 | 602.9 ± 771.6 | 0.825 |
| Potassium, mmol/l | 83.1 ± 31.8 | 78.2 ± 33.9 | 0.379 | 87.8 ± 34.1 | 85.6 ± 36.1 | 0.788 |
| Creatinine, mg/dl | 40.5 ± 20.7 | 42.1 ± 21.4 | 0.569 | 45.0 ± 22.4 | 40.4 ± 21.1 | 0.105 |

**Abbreviations;** SBP, systolic blood pressure; DBP, diastolic blood pressure; BMI, body mass index; HDL-C, high density lipoprotein cholesterol; LDL-C, low density lipoprotein cholesterol; PTH, parathyroid hormone; BUN, blood urea nitrogen; eGFR, estimated glomerular filtration rate; CKD epidemiology collaboration equation, CKD-EPI; MDRD, modification of diet in renal disease equation

**Supplementary Table 4. Mean differences in daily nutritional status according to the diet**.

|  | Differences  within the  MEDi-POB diet | Differences  within the control  diet | Differences  between the  control diet and  MEDi-POB diet | *p1* | *p2* | *p3* |
| --- | --- | --- | --- | --- | --- | --- |
| MDS | 4.93(4.43,5.44) | -0.21(-0.71,0.29) | -5.14(-5.81,-4.48) | * | - | * |
| Q1 | 0.41(0.25,0.58) | -0.01(-0.17,0.16) | -0.42(-0.64,-0.21) | - | - | * |
| Q2 | 0.59(0.46,0.72) | 0.05(-0.08,0.18) | -0.54(-0.72,-0.36) | - | * | * |
| Q3 | 0.93(0.84,1.02) | 0.04(-0.06,0.13) | -0.90(-1.03,-0.77) | - | - | * |
| Q4 | 0.16(0.01,0.30) | 0.14(-0.01,0.28) | -0.02(-0.21,0.17) | - | - | - |
| Q5 | 0.24(0.10,0.37) | -0.02(-0.16,0.12) | -0.26(-0.45,-0.06) | - | - | * |
| Q6 | 0.04(-0.04,0.13) | 0.06(-0.02,0.14) | 0.02(-0.10,0.13) | - | - | - |
| Q7 | 0.29(0.15,0.42) | 0.14(0.00,0.28) | -0.15(-0.34,0.05) | - | - | - |
| Q8 | - | - | - | - | - | - |
| Q9 | 0.07(-0.08,0.22) | -0.04(-0.19,0.10) | -0.11(-0.31,0.08) | - | * | - |
| Q10 | 0.92(0.83,1.00) | -0.02(-0.11,0.06) | -0.94(-1.06,-0.82) | - | - | * |
| Q11 | 0.33(0.16,0.49) | -0.08(-0.25,0.09) | -0.41(-0.63,-0.18) | - | - | * |
| Q12 | 0.44(0.27,0.60) | 0.01(-0.16,0.17) | -0.43(-0.66,-0.20) | - | - | * |
| Q13 | -0.05(-0.15,0.06) | -0.06(-0.17,0.04) | -0.02(-0.13,0.10) | - | - | - |
| Q14 | 0.57(0.42,0.73) | -0.40(-0.55,-0.25) | -0.97(-1.16,-0.79) | - | - | * |

Abbreviations: MDS, Mediterranean diet score

Q1: 1 point for using perilla oil or olive oil as the principal cooking fat; Q2: 1 point for consuming ≥3 teaspoons of perilla oil or olive oil per day; Q3: 1 point for consuming ≥2 servings of vegetables per day; Q4: 1 point for consuming ≥1 piece of fruit per day; Q5: 1 point for consuming <1 serving of red meat or sausages per day; Q6: 1 point for consuming <1 serving of butter, margarine, or cream per day (1 serving = 1 teaspoon); Q7: 1 point for consuming <1 serving of sugar-sweetened beverages per day; Q8: 1 point for consuming ≥7 servings of wine per week; Q9: 1 point for consuming ≥3 servings of beans or tofu per week; Q10: 1 point for consuming ≥3 servings of fish or seafood per week; Q11: 1 point for consuming <3 servings of sweets, non-whole wheat bread, cakes, or cookies per week; Q12: 1 point for consuming ≥3 servings of nuts per week; Q13: 1 point for preferring white meat over red meat; Q14: 1 point for consuming ≥3 servings of whole grains per week.

**Supplementary Table 5. Nutritional status at baseline and after intervention based on the MEDi-POB diet and control diet.**

|  | Medi-POB diet |  |  | Control diet |  |  |
| --- | --- | --- | --- | --- | --- | --- |
|  | Pre | Post | P-value | Pre | Post | P-value |
| MDS score | 5.3 ± 1.7 | 10.2 ± 1.1 | <0.001 | 5.0 ± 1.8 | 4.9 ± 1.4 | 0.468 |
| Total calorie, kcal/day | 1191.0 ± 405.1 | 1437.7 ± 413.5 | 0.013 | 1307.8 ± 584.5 | 1212.6 ± 417.6 | 0.221 |
| Carbohydrate, g | 178.5 ± 68.0 | 200.6 ± 55.9 | 0.117 | 188.7 ± 67.5 | 178.3 ± 60.7 | 0.334 |
| Fat, g | 31.7 ± 16.7 | 50.2 ± 21.0 | <0.001 | 36.8 ± 36.0 | 32.0 ± 20.5 | 0.352 |
| Protein, g | 42.8 ± 21.1 | 48.1 ± 15.8 | 0.245 | 50.0 ± 27.2 | 48.7 ± 25.1 | 0.779 |
| Carbohydrate, % | 60.3 ± 11.2 | 56.7 ± 8.5 | 0.064 | 60.4 ± 13.2 | 60.5 ± 13.2 | 0.962 |
| Fat, % | 23.6 ± 7.6 | 30.5 ± 7.4 | <0.001 | 22.7 ± 11.0 | 22.6 ± 9.8 | 0.968 |
| Protein, % | 14.3 ± 3.9 | 13.4 ± 2.4 | 0.117 | 15.4 ± 5.4 | 15.6 ± 4.5 | 0.868 |
| Fiber, g | 14.5 ± 6.3 | 20.8 ± 9.1 | <0.001 | 14.7 ± 6.9 | 13.7 ± 6.5 | 0.952 |
| Vitamin A, ug RAE | 237.6 ± 170.4 | 264.7 ± 105.2 | 0.553 | 268.1 ± 234.4 | 285.4 ± 333.0 | 0.763 |
| Retinol, ug | 70.5 ± 84.9 | 30.5 ± 24.8 | 0.002 | 75.8 ± 107.4 | 79.5 ± 79.8 | 0.845 |
| β-carotene, ug | 1977.5 ± 1854.2 | 1742.4 ± 923.7 | 0.266 | 2326.9 ± 2716.4 | 2539.2 ± 3559.7 | 0.744 |
| Vitamin E, mg | 10.6 ± 6.1 | 10.7 ± 3.2 | 0.846 | 10.2 ± 6.7 | 10.1 ± 6.0 | 0.896 |
| Vitamin K, ug | 107.8 ± 133.0 | 98.5 ± 59.0 | 0.415 | 128.7± 132.5 | 114.1 ± 229.3 | 0.716 |
| Vitamin C, mg | 59.0 ± 52.3 | 90.4 ± 47.6 | 0.003 | 61.9 ± 50.7 | 69.8 ± 81.8 | 0.523 |
| Thiamin, mg | 1.07 ± 0.48 | 0.94 ± 0.42 | 0.077 | 1.22 ± 0.90 | 1.17 ± 0.68 | 0.713 |
| Riboflavin, mg | 0.84 ± 0.50 | 0.93 ± 0.33 | 0.480 | 0.86 ± 0.52 | 0.94 ± 0.58 | 0.391 |
| Niacin, mg | 7.3 ± 3.9 | 10.7 ± 3.7 | 0.002 | 9.4 ± 8.7 | 8.3 ± 5.0 | 0.400 |
| Vitamin B12, ug | 4.5 ± 5.8 | 1.7 ± 2.6 | 0.005 | 6.8 ± 9.1 | 5.3 ± 7.3 | 0.155 |
| Calcium, mg | 279.9 ± 191.3 | 292.4 ± 134.2 | 0.807 | 254.3 ± 120.9 | 268.8 ± 130.4 | 0.552 |
| Phosphorus, mg | 631.5 ± 271.2 | 629.9 ± 201.4 | 0.784 | 674.2 ± 315.0 | 669.1 ± 279.5 | 0.923 |
| Sodium, mg | 2078.3 ± 1270.1 | 1390.2 ± 785.0 | 0.002 | 2456.7 ± 1054.3 | 2300.2 ±1296.4 | 0.434 |
| Potassium, mg | 1489.5 ± 587.0 | 1615.8 ± 465.7 | 0.263 | 1719.6 ± 718.6 | 1548.0 ± 588.1 | 0.072 |
| Magnesium, mg | 63.3 ± 36.0 | 49.9 ± 27.3 | 0.013 | 79.5 ± 42.4 | 64.2 ± 40.4 | 0.020 |
| Iron, mg | 10.4 ± 5.3 | 8.3 ± 2.7 | 0.004 | 11.2 ± 6.8 | 10.2 ± 6.1 | 0.498 |
| Zinc, mg | 6.1 ± 2.8 | 5.2 ± 3.0 | 0.080 | 6.8 ± 3.9 | 7.2 ± 4.0 | 0.542 |
| Cupper, ug | 407.3 ± 216.9 | 278.2 ± 180.6 | 0.001 | 427.2 ± 275.1 | 392.3 ± 244.3 | 0.350 |
| Cholesterol, mg | 193.9 ± 210.8 | 153.2 ± 90.2 | 0.184 | 186.5 ± 213.3 | 224.8 ± 239.8 | 0.261 |
| Saturated fat, g | 6.7 ± 4.0 | 7.9 ± 2.8 | <0.001 | 7.7 ± 7.4 | 7.6 ± 7.4 | 0.970 |
| Monounsaturated fat, g | 8.0 ± 4.9 | 14.8 ± 7.3 | 0.108 | 8.2 ± 9.7 | 8.2 ± 9.0 | 0.972 |
| Polyunsaturated fat, g | 9.2 ± 7.6 | 13.8 ± 6.1 | <0.001 | 7.1 ± 6.1 | 6.5 ± 4.4 | 0.525 |
| N-3 PUFA, g | 0.6 ± 0.9 | 1.4 ± 0.8 | 0.001 | 0.6 ± 0.9 | 0.3 ± 0.4 | 0.090 |
| N-6 PUFA, g | 4.0 ± 5.5 | 9.3 ± 5.4 | <0.001 | 2.4 ± 2.9 | 2.2 ± 2.8 | 0.730 |

**Abbreviations;** SBP, systolic blood pressure; DBP, diastolic blood pressure; BMI, body mass index; HDL-C, high density lipoprotein cholesterol; LDL-C, low density lipoprotein cholesterol; PTH, parathyroid hormone; BUN, blood urea nitrogen; eGFR, estimated glomerular filtration rate; CKD epidemiology collaboration equation, CKD-EPI; MDRD, modification of diet in renal disease equation

**Supplementary Table 6. Baseline cytokines and myokines status study population**

|  | Group1 | Group2 | p-value |
| --- | --- | --- | --- |
| Cytokines |  |  |  |
| Cytokine GM-CSF | 14.4 ± 5.9 | 13.8 ± 5.1 | 0.567 |
| Cytokine INF-r | 2.0 ± 4.7 | 4.6 ± 12.9 | 0.243 |
| Cytokine IL-1b | 12.6 ± 11.8 | 9.7 ± 5.7 | 0.149 |
| Cytokine IL-10 | 7.8 ± 3.0 | 8.8 ± 4.0 | 0.02 |
| Cytokine IL-12p70 | 38.6 ± 30.1 | 50.7 ± 36.9 | 0.055 |
| Cytokine IL-2 | 11.0 ± 5.3 | 11.9 ± 6.2 | 0.102 |
| Cytokine IL-4 | 111.6 ± 38.5 | 109.4 ± 46.1 | 0.607 |
| Cytokine IL-5 | 4.5 ± 1.4 | 5.0 ± 2.0 | 0.015 |
| Cytokine IL-6 | 11.7 ± 4.4 | 12.3 ± 5.3 | 0.443 |
| Cytokine IL-8 | 120.2 ± 599.3 | 33.3 ± 17.9 | 0.329 |
| Cytokine TNF-a | 59.4 ± 10.6 | 61.0 ± 11.1 | 0.369 |
| Cytokine VEGF | 98.3 ± 95.8 | 86.3 ± 44.7 | 0.255 |

.
